# Rewiring ALS by modulating the autophagy receptor SQSTM1

**DOI:** 10.1016/j.stemcr.2026.102976

**Published:** 2026-06-25

**Authors:** Laetitia Aubry, Viktor I. Korolchuk, Sovan Sarkar

**Affiliations:** 1 CECS/AFM, I-STEM, Corbeil-Essonnes 91100, France; 2 INSERM/UEPS, UMR 861, Paris-Saclay University, I-STEM, Corbeil-Essonnes 91100, France; 3 Biosciences Institute, Faculty of Medical Sciences, Newcastle University, Newcastle upon Tyne NE4 5PL, UK; 4 Department of Cancer and Genomic Sciences, School of Medical Sciences, College of Medicine and Health, University of Birmingham, Birmingham B15 2TT, UK

## Abstract

Drug screening for genetic disorders is limited by difficulty identifying disease-relevant phenotypes. In this issue, Roussange et al., show that reverse phenotypic mapping could uncover therapeutic gene expression signatures. Using this approach, they identified prazosin, which increases SQSTM1 expression and rescues disease phenotypes in iPSC-derived motor neurons and zebrafish model of amyotrophic lateral sclerosis with SQSTM1 haploinsufficiency.

## Teaser

Drug screening for genetic disorders is limited by difficulty identifying disease-relevant phenotypes. Roussange et al., in this issue, demonstrated that reverse phenotypic mapping could uncover therapeutic gene expression signatures. They identified prazosin, boosting SQSTM1 expression and rescuing zebrafish and induced pluripotent stem cell (iPSC)-derived motor neuron models of amyotrophic lateral sclerosis with SQSTM1 haploinsufficiency.

## Main text

The classical approach of drug discovery relies on compound screening against predefined molecular targets or phenotypic readouts linked to a specific human disease. This strategy, exemplified by forward pharmacology approaches, aims to identify drug candidates capable of rescuing the chosen disease-relevant phenotype in cell or animal models of the disease. Such reductionist framework related to target- or phenotype-based drug discovery, where modulation of a single biological target or process is exploited to treat diseases, has led to the discovery of various clinically used drugs. However, therapeutic discovery in these scenarios is limited due to the complexity of human disorders driven by widespread perturbations in gene expression and cellular processes and the inability to account for broader biological context including the full spectrum of molecular alterations and cellular heterogeneity (Vincent et al., 2022).

In this issue, Roussange et al. adopted an alternative approach of reverse phenotypic mapping to identify therapeutic drug candidates (Roussange et al., 2026). This strategy involves screening of compounds by generating their gene expression signatures and then mapping against the gene expression profiles of many diseases to identify drugs that can reverse the disease state back to normal healthy state. Existing resources such as the Broad Institute’s Connectivity Map have shown the power of linking drug-induced gene expression signatures in various tumor cell types to disease-associated molecular profiles, enabling the identification of compounds for drug development against cancers (Subramanian et al., 2017). Moreover, research undertaken in disease-relevant cell types and combined with drug repurposing strategies, which leverage existing medications with established safety profiles, have the potential to accelerate therapeutic development and downstream clinical translation. Such an approach offers significant advantage to develop therapeutics especially for monogenic disorders, as demonstrated in this study for a rare form of a neurodegenerative disorder called amyotrophic lateral sclerosis (ALS) for which there is currently no cure (Roussange et al., 2026).

The study utilized human embryonic stem cell (hESC)-derived mesodermal progenitor cells to generate gene expression signatures of 50 biologically active compounds, which were selected based on their high clinical use, known safety profiles, and broad chemical and biological diversity (Roussange et al., 2026). The cellular platform was chosen because of its ease of production, cost effectiveness, and established suitability for drug screening applications. Drug treatment was performed at 10 μM concentration for 24 h duration under conditions that did not induce overt cytotoxicity, similar to the experimental paradigm used in the Broad Institute’s Connectivity Map study. The compounds were evaluated by transcriptomics analysis using next-generation sequencing for their ability to regulate transcript expression and alternative splicing. While majority of the compounds either induced modest splicing alterations targeting genes associated with genetic disorders or caused minimal transcriptional changes, no direct therapeutic benefit could be linked to the exon-skipping changes arising from clinically relevant genetic variants. However, more than one-quarter of the differentially expressed genes were found to be mutated in monogenic diseases. Among the validated hits in hESC-derived mesodermal progenitor cells, prazosin was identified as a modulator of *SQSTM1*, which is associated with frontotemporal dementia and ALS3 (FTD/ALS3) (Fecto et al., 2011; Roussange et al., 2026). Prazosin-induced upregulation of *SQSTM1* expression was further confirmed by RT-qPCR analysis in hESC-derived motor neurons, which are the primary disease-affected cell type in ALS (Roussange et al., 2026). This regulatory interaction was not robustly detected in the Broad Institute Connectivity Map database that was generated using various cancer cell lines, thereby highlighting the importance of disease-relevant human stem cell models for identifying cell type-specific therapeutic mechanisms.

SQSTM1, also known as sequestosome-1 or p62, is a multifunctional receptor protein that plays a central role in autophagy, which is an intracellular quality-control pathway essential for maintaining cellular homeostasis and survival (Rogov et al., 2014). Autophagy is responsible for the removal of undesirable cellular materials, including protein aggregates and damaged organelles. This process involves the formation of autophagosomes and their subsequent fusion with the lysosomes to form autolysosomes, where the autophagic cargo is degraded. As a key autophagy receptor, SQSTM1 recognizes polyubiquitinated protein aggregates through its ubiquitin-associated (UBA) domain and links them to LC3-positive autophagosomes via its LC3-interacting region (LIR) domain, thereby facilitating their selective degradation (Rogov et al., 2014). In addition, SQSTM1 can undergo liquid-liquid phase separation, enabling the sequestration of toxic protein aggregates into dense condensates that promote efficient autophagic clearance (Kurusu et al., 2024).

Loss-of-function mutations in *SQSTM1* lead to haploinsufficiency in FTD/ALS3 and are associated with impairment in selective autophagy pertaining to the recognition and clearance of ubiquitinated autophagic cargo (Deng et al., 2020). In patients’ fibroblasts harboring heterozygous mutations in *SQSTM1*, the screen hit prazosin elevated SQSTM1 at both transcript and protein levels. The effect of prazosin on SQSTM1 upregulation was accompanied by concomitant induction of autophagy in FTD/ALS3 patient’s fibroblasts, suggesting that the restoration of SQSTM1 under haploinsufficient conditions may rescue autophagic function (Roussange et al., 2026). Prazosin is an anti-hypertensive drug used to lower high blood pressure by acting as an α_1_-adrenergic receptor antagonist (Perez, 2023). Although other α_1_-adrenergic blockers of the same chemical family have shown neuroprotective effects in ALS models (Carroll et al., 2025), these compounds did not influence the SQSTM1 levels (Roussange et al., 2026). This suggests that the mechanism of action of prazosin in upregulating SQSTM1 is independent of the α-adrenergic receptor blockade.

For disease modeling and drug testing in relevant cellular platform, CRISPR-Cas9-mediated genome editing was performed in human induced pluripotent stem cells (hiPSCs) to generate SQSTM1 haploinsufficient (*SQSTM1*^*+/−*^) and knockout (*SQSTM1*^*−/−*^) lines where the parental hiPSC line served as an isogenic control (*SQSTM1*^*+/+*^). The genome-edited hiPSC lines retained pluripotency and genomic integrity, displayed no detectable off-target effects, and were successfully differentiated into spinal motor neurons that are affected in ALS. Loss or depletion of SQSTM1 in hiPSC-derived motor neurons was associated with impairment in autophagic activity and trafficking of protein aggregates to aggresomes for subsequent clearance by autophagy (Roussange et al., 2026). Notably, the SQSTM1-deficient or -depleted motor neurons exhibited disruption of neurite network integrity, which has been reported in neuronal cell models of ALS. These cellular phenotypes underscore a critical role of SQSTM1 in maintaining motor neuron homeostasis.

Of biomedical relevance, prazosin upregulated SQSTM1 in hiPSC-derived motor neurons with *SQSTM1* knockdown. Restoration of SQSTM1 by prazosin was associated with autophagy induction and rescue of the neuritic phenotype in these neuronal cells with SQSTM1 haploinsufficiency (Roussange et al., 2026). Furthermore, prazosin increased SQSTM1 levels *in vivo* in a zebrafish model of ALS with *sqstm1* knockdown. In this ALS zebrafish model, prazosin treatment improved motor deficits and swimming behavior without detectable toxicity. These beneficial effects were not recapitulated by alfuzosin, another α_1_-adrenergic receptor antagonist that does not regulate *SQSTM1*, suggesting that the therapeutic effects of prazosin are linked to SQSTM1 upregulation, rather than α_1_-adrenergic receptor blockade alone (Roussange et al., 2026). These findings support prazosin as a promising candidate for drug repurposing in ALS associated with SQSTM1 haploinsufficiency.

Beyond restoring haploinsufficient gene expression, prazosin also enhances autophagic activity that represents an important cellular benefit to the brain. Autophagy is vital for neuronal homeostasis and survival, and its dysfunction is a common pathogenic mechanism across various neurodegenerative conditions. Defective autophagy has been reported in familial ALS associated with pathogenic mutations in *SOD1* (superoxide dismutase 1), *C9orf72* (chromosome 9 open reading frame 72), *TARDBP* (TAR DNA-binding protein, encoding TDP-43), *OPTN* (optineurin), and *SQSTM1*, amongst others (Seranova et al., 2020). Therefore, restoring functional autophagic activity for the clearance of unwanted cellular materials, including aggregated proteins and damaged mitochondria, is critical for preserving neuronal health. Indeed, several studies have demonstrated the beneficial effects of pharmacological autophagy inducers in transgenic animal models and patient iPSC-derived neuronal cells of both common and rare neurodegenerative diseases, including ALS (Seranova et al., 2020).

This study highlights the utility of human pluripotent stem cell-based models for reverse phenotypic drug discovery, enabling the identification of candidate therapeutics and the subsequent demonstration of their efficacy in disease-relevant human cell type and animal models (Roussange et al., 2026). While the drug-induced molecular changes have been shown to rescue the disease-relevant phenotypes in ALS models, further work is warranted to elucidate the precise mechanisms by which prazosin regulates *SQSTM1* expression and autophagy. Because prazosin also regulated other autophagy, endocytic, and lysosomal genes, one possibility could be investigating any underlying role of TFEB (transcriptional factor EB) and other MiT-TFE (microphthalmia-transcription factor E) family transcription factors that govern autophagy and lysosomal gene expression. Moreover, because prazosin is a lysomotrophic agent, a deeper understanding of its effects on lysosomal properties and function is needed in the context of ALS. Beyond the experimental model systems used in this study, prazosin could be tested in patient iPSC-derived brain organoid and mouse models of ALS. Despite multiple aspects of further work to be conducted, this study paves the way for future integration of human stem cell-derived cellular models with artificial intelligence-driven analytics as a powerful platform for drug discovery, particularly for rare monogenic disorders lacking effective models and therapies.

## Acknowledgments

The authors acknowledge funding support from the Medical Research Council (MR/Z504488/1) to S.S. and V.I.K.; Action Medical Research/LifeArc (GN3049), LifeArc (P2019-0004), and Wolfram Syndrome UK to S.S.; BBSRC AGENT Network (BB/W018381/1) and VitaDAO/Molecule and Procter & Gamble academic partnerships to V.I.K.; and Laboratoire d’Excellence Revive (Investissement d’Avenir; ANR-10-LABX-73), and the Region Ile-de-France via doctoral school Innovation Thérapeutique, du Fondamental à l’Appliqué (ED 569) from Université Paris-Saclay to L.A. I-Stem/CECS is part of the Biotherapies Institute for Rare Diseases (BIRD) supported by the Association Française contre les Myopathies (AFM-Téléthon). Both S.S. and V.I.K. are Former Fellows for Life at Hughes Hall, University of Cambridge, United Kingdom.

## Declaration of interests

S.S. is a scientific advisor for NMN Bio Ltd.; V.I.K. is a scientific advisor for Longaevus Technologies.

## Declaration of generative AI and AI-assisted technologies in the writing process

During the preparation of this work, the authors used ChatGPT from OpenAI in order to improve readability and language. After using this tool, the authors reviewed and edited the content as needed and take full responsibility for the content of the publication.
